# Microfluidics for COVID-19: From Current Work to Future Perspective

**DOI:** 10.3390/bios13020163

**Published:** 2023-01-20

**Authors:** Qi Li, Xingchen Zhou, Qian Wang, Wenfang Liu, Chuanpin Chen

**Affiliations:** 1 Department of Pharmacy, Xiangya School of Pharmaceutical Sciences, Central South University, Changsha 410017, China; 2 Department of Pharmacy, Xiangya Hospital, Central South University, Changsha 410017, China

**Keywords:** microfluidic, COVID-19, molecular assays, immunoassays

## Abstract

Spread of coronavirus disease 2019 (COVID-19) has significantly impacted the public health and economic sectors. It is urgently necessary to develop rapid, convenient, and cost-effective point-of-care testing (POCT) technologies for the early diagnosis and control of the plague’s transmission. Developing POCT methods and related devices is critical for achieving point-of-care diagnosis. With the advantages of miniaturization, high throughput, small sample requirements, and low actual consumption, microfluidics is an essential technology for the development of POCT devices. In this review, according to the different driving forces of the fluid, we introduce the common POCT devices based on microfluidic technology on the market, including paper-based microfluidic, centrifugal microfluidic, optical fluid, and digital microfluidic platforms. Furthermore, various microfluidic-based assays for diagnosing COVID-19 are summarized, including immunoassays, such as ELISA, and molecular assays, such as PCR. Finally, the challenges of and future perspectives on microfluidic device design and development are presented. The ultimate goals of this paper are to provide new insights and directions for the development of microfluidic diagnostics while expecting to contribute to the control of COVID-19.

## 1. Introduction

Severe acute respiratory syndrome coronavirus 2 (SARS-CoV-2) has triggered the global spread of coronavirus disease 2019 (COVID-19) [1,2], and the World Health Organization reported that, by August 2022, more than 620 million infections and more than 6.5 million deaths had been confirmed worldwide [3]. Characteristics such as a rapid and widespread transmission, an uncertain incubation period, and nonspecific symptoms are why this outbreak is challenging to control [4,5,6]. Furthermore, as the epidemic progressed, the genetic material of the virus was altered, which affected the rate of transmission, the degree of symptoms after infection, and the effectiveness of drugs and vaccines, ultimately making the epidemic more difficult to control [7]. So far, the epidemic has lasted for nearly three years, during which the blow to both global public health and the economy has been tremendous. On the one hand, the rampant spread of the virus has posed a severe threat to human life and health. On the other hand, the shutdown and repeated closures to control the epidemic have seriously slowed down the global economy; thus, it is worth thinking about how to prevent and control COVID-19 and promote economic recovery effectively.

There are three main ways to control COVID-19 as an infectious disease: controlling the source of the infection, cutting off the channels of transmission, and protecting susceptible populations [8,9,10,11,12]. Among these approaches, the first one is considered the most important. The main sources of infection of COVID-19 are confirmed cases and asymptomatic patients, and the early detection, isolation, and treatment of these patients is necessary to effectively control the infectious source. However, the uncertain incubation period and the presence of asymptomatic patients make this initiative very difficult; therefore, it is of great importance to develop rapid, sensitive, and specific field detection methods and kits [13]. COVID-19 detection is divided into two main categories: molecular detection methods for detecting nucleic acids of SARS-CoV-2 and immunoassays for detecting antibodies or antigens [14]. Molecular detection methods usually use respiratory samples, such as nasal or pharyngeal swabs, under laboratory conditions, and the most commonly used method is reverse transcription polymerase chain reaction (RT-PCR). Isothermal nucleic acid amplifications have also been widely studied due to the fact of their advantages of no thermal cycler, simplicity, and rapidity, which make them more suitable for field detection. In immunoassays, antigen detection is often performed using nasal swabs or pharyngeal swabs as a complementary method to nucleic acid detection, while antibody detection is performed using serum or whole blood as a sample, mainly as a classification and screening tool for IgM/IgG potency [15]. Both molecular detection and immunoassays require specialized personnel in the laboratory, which is time consuming, and they require complex instruments and professional operation. In this context, the market demand and outstanding advantages of point-of-care testing (POCT) products have led to the rapid development of this industry. The ideal POCT system should be user friendly, easy to use, and low cost, with high clinical sensitivity, specificity, and accuracy, and, more importantly, provide immediate results [16], which would make them suitable for large-scale immediate diagnosis, enable rapid detection, and provide timely results in primary hospitals and communities for early diagnosis, isolation, and treatment.

Microfluidics is a powerful technology for the realization of POCT diagnosis, which can integrate and miniaturize the multiple steps involved in sample detection on a small chip. Furthermore, due to the small reaction chamber of the chip, the consumption of detection reagents and sample volume is also small, which can effectively reduce costs. In addition, microfluidic chips have the advantages of high throughput and less contamination. The combination of microfluidic technology with traditional laboratory assays is expected to lead to automated POCT, monitoring of COVID-19 disease transmission in a potential home diagnostic model, and provide new solutions for both developed and developing countries [17].

In this review, according to the different driving forces of the fluid, we introduce the common POCT devices based on microfluidic technology on the market, including paper-based microfluidic (μPADs), centrifugal microfluidic, optical fluid, and digital microfluidic platforms. Furthermore, various microfluidic-based diagnostic COVID-19 assays, including immunoassay methods, such as enzyme-linked immunosorbent assay (ELISA) and nucleic acid detection methods (e.g., PCR), are discussed. Finally, the challenges of and future perspectives on microfluidic platform design and development are presented. We hope that this paper will provide new insights into the development of microfluidic diagnostics while contributing to the control of COVID-19.

## 2. Microfluidic Platform

A microfluidic chip, also known as an LOC, is a mainstream technology for next-generation POCT [18,19,20], with easy integration, high-speed analysis, low energy consumption, low physical consumption, and low contamination. An LOC normally consists of a chip, an analyzer, a drive source, a signal detection device, and a functionalized kit. A microfluidic chip has the following advantages: First, the chip can significantly reduce reagent consumption by reducing the detection volume. Secondly, with the decrease in the characteristic scale, the surface tension (capillary force, etc.) plays a leading role in the liquid motion, which makes passive liquid propulsion detection methods such as test strips possible. Thirdly, the laminar flow with a low Reynolds number can produce a more stable liquid–liquid interface, which lays the foundation for a higher sensitivity.

Based on the microfluidic manufacturing materials, we can divide the chips into paper-based, glass, silicon, and polymer types (commonly used: polymethyl methacrylate, polystyrene, polycarbonate, and PDMS). In the fluid driving, centrifugal force, electrochemical pumping, capillary action, manual fluid transfer, hydrostatic pressure, isokinetic electrophoresis, and injection pump are all commonly used liquid operation strategies. Finally, according to the characteristics and functional positioning of the microfluidic chip, we can also divide the microfluidic platform into paper-based microfluidic, centrifugal microfluidic, digital droplet microfluidic, high-throughput microfluidic, multiplex microfluidic, and fully automatic microfluidic.

**Figure 1:**
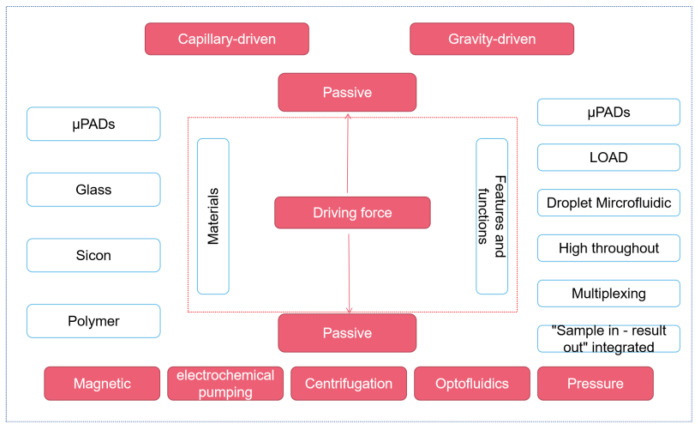
Different microfluidic platforms classified according to the driving force.

According to the different driving forces of fluid, this chapter mainly introduces the microfluidic platform in two parts: passive control and active control (as shown in Figure 1). In passive manipulation, we focus on μPADs based on the capillary driving force, while in active manipulation, we mainly introduce the centrifugal microfluidic, optical fluidic, and digital microfluidic platforms.

### 2.1. Passive Microfluidic Platform—μPADs

Passive microfluidic technology can be divided into capillary driving and gravity driving. Passive microfluidic technology has many unique advantages. Firstly, passive microfluidic platforms are usually easier to make and easier to expand production. Secondly, a passive microfluidic platform is usually easier to operate and can conduct self-tests at home without the operation of professional technicians. Thirdly, compared with an active microfluidic platform, the passive microfluidic platform does not need an external power supply, so the cost is lower, and it is undoubtedly a promising platform for areas with limited resources.

μPADs are a representative passive microfluidic platform, which use patterned paper as the carrier and distributes liquid through capillarity. μPADs are suitable for multiplex assays of various samples and are compatible with various assays. In contrast to traditional lateral flow strips, μPADs can autonomously drive multistep sequences, while the timing of the reagent’s movement can be controlled by adjusting the length and fluid volume of each channel [21]. Compared to traditional microfluidic labs, using paper sheets as a carrier also has unique advantages. Firstly, paper sheets have good biocompatibility, easy absorption of reagents, and are suitable for clinical analysis. Secondly, the inherent white background of paper sheets can provide contrast for color-based methods. Thirdly, paper is an ideal material for manufacturing environmentally friendly portable equipment because of its lightweight and low cost. Based on these advantages, μPADs have received attention in POCT applications, with rapid POCT detection of infectious diseases, especially in resource-poor areas, being one of the most popular areas. Herein, the following will discuss the development of μPADs for COVID-19 POCT.

Some researchers have made great efforts in the system integration, portability, and operation procedure simplification of paper-based microfluidic analysis. Li et al. designed a paper-based electrochemical impedance sensor by synthesizing ZnO nanowires directly on the working electrode by the hydrothermal growth method on the μPADs [22]. This paper-based electrochemical impedance spectroscopy solves the problems of the paper-based ELISA method that requires labeling and extra steps to amplify the signal but retains the advantages of the simple fabrication and low cost of μPADs, and the whole process can be completed within 30 min. It is simple, environmentally friendly, and fast, making COVID-19 antibody testing easier.

**Figure 2:**
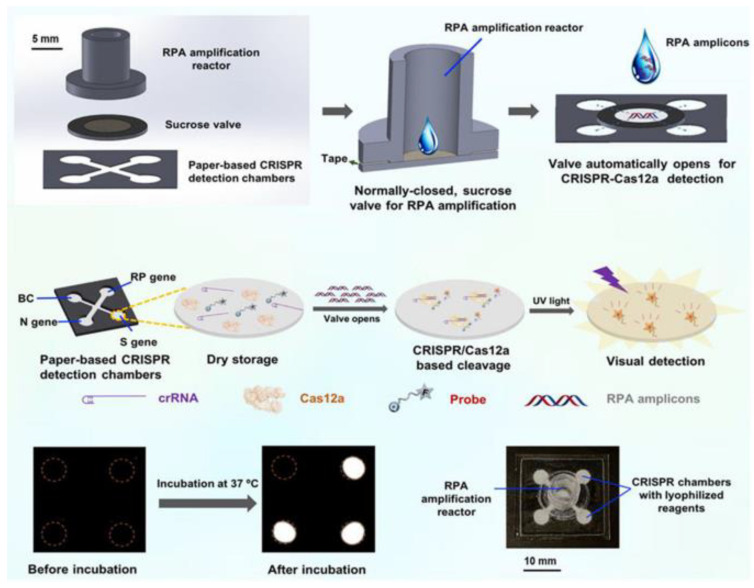
Schematic diagram of paper-based microfluidics for SARS-CoV-2 detection. Reproduced with permission from Li, X., *Biosens. Bioelectron.*, published by Elsevier, 2021 [23].

In addition, some researchers have worked hard to improve the automation and sensitivity of the detection method. Yin et al. designed an automated paper-based microfluidic multiplex detection platform that combines clustered regularly interspaced short palindromic repeats (CRISPR) technology and recombinase polymerase amplification (RPA) to detect COVID-19, with a total detection time of less than 1 h and a sensitivity of 100 copies, which is comparable to RT-PCR (Figure 2) [23]. The paper-based microfluidic chip’s CRISPR detection chamber is designed and fabricated using wax-printing technology. The printed RPA reaction chamber is connected to the CRISPR detection chamber using a programmable, normally closed paper-based sucrose valve, which automatically opens when a preset time is reached. At this time, the RPA amplicon migrates to the CRISPR detection chamber, enabling the automatic transfer of products, and carry-over contaminations are reduced. Furthermore, the μPADs can be stored at room temperature for 30 days without needing cold chain transport, significantly improving the feasibility of detecting SARS-CoV-2 infection in the field. In conclusion, this experimental platform enables simple, low-cost, and automated multigene detection and is a meaningful research attempt to detect COVID-19 in resource-limited areas.

Compared with traditional microfluidic platforms, μPADs are more convenient to use, less expensive to manufacture, simple, and nonpolluting for post-processing. They can be performed by simple and safe combustion, but using paper as a carrier provides benefits but also has some limitations. For some samples with low surface tension, the hydrophobic zone is not necessarily hydrophobic enough and may be prone to leakage; in addition, samples tend to remain in the paper channel leading to low sample utilization; most importantly, the paper chip is still lacking in the issue of sensitivity.

### 2.2. Active Microfluidic Platforms

#### 2.2.1. Centrifugal Microfluidic Platform

**Figure 3:**
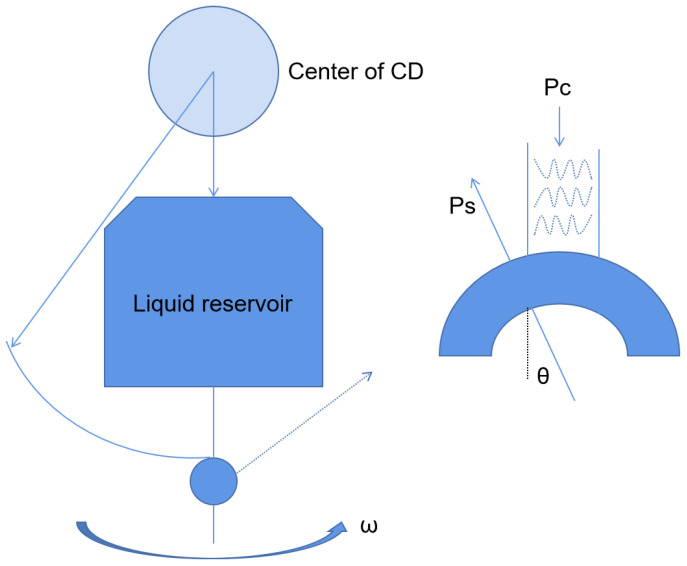
Schematic diagram of the centrifugal chip principle.

Centrifugal microfluidics is a kind of microfluidic system that uses centrifugal force as the driving force of the liquid flow to realize reagent detection and analysis. Centrifugal microfluidics has many advantages. First, centrifugal forces are present on all surfaces of the disk (Figure 3), so liquid transport is simple and efficient. Secondly, the physical and chemical properties of the sample have little effect on the microfluidic panel, which is good for body fluid samples. Thirdly, centrifugal microfluidics can be highly integrated. The whole test process, such as sample pretreatment, mixing, and valve control, can be realized on a single disk [24,25]. For example, Malic et al. fabricated a disk for the rapid detection of SARS-CoV-2 from whole blood based on a centrifugal microfluidic platform [26]. The priming enzyme and reaction buffer were prefixed in the reaction chamber, and the disk could be rotated in different directions by the spindle motor to move the solution to the waste chamber or collection chamber for purification, amplification, and detection. The device was subsequently shown to successfully detect SARS-CoV-2 in whole blood samples from purified samples to result in a limit of detection (LOD) as low as 0.5 copies/uL. Moreover, the platform could complete the process in less than 60 min. All these device characteristics demonstrate its potential for the diagnosis of SARS-CoV-2 by POCT in resource-poor areas.

Centrifugal microfluidics has great advantages in sample mixing, step simplification, and high integration, but in order to further promote it, efforts need to be made to reduce the volume of equipment and reduce the use of external equipment. Dignan et al. developed the first centrifugal microdevice to prepare high-purity nucleic acids comprehensively from virgin oral swab samples [27]. Unlike previous centrifugal microfluidic devices, this system automatically uses controllable laser-on-board microvalves instead of passive valves sensitive to temperature fluctuations and changes in channel surface energy, providing better reproducibility under various environmental conditions and achieving tight spatiotemporal control of the fluid flow. In this study, centrifugal microfluidic technology was combined with dynamic solid-phase extraction to achieve on-disk extraction of DNA or RNA and with loop-mediated isothermal amplification (LAMP) technology to fabricate an automatable, portable microfluidic platform-based nucleic acid preparation device, which allows for its practical use in the field by nontechnical personnel.

#### 2.2.2. Optical Fluid Microfluidic Platform

**Figure 4:**
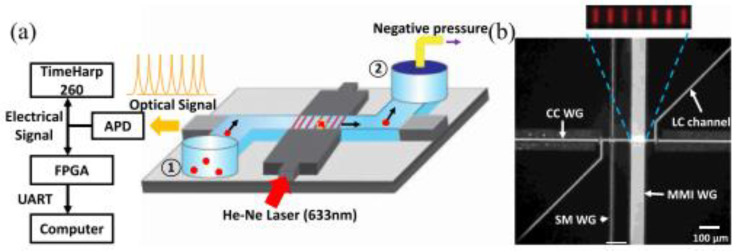
FPGA analysis platform based on ARROW optical flow control device. (**a**) Schematic diagram of devices used for fluorescence signal acquisition and analysis. (**b**) Optical microscope image of ARROW optical fluidic device. Reproduced with permission from Mohammad, J, N, S., *IEEE Photonics J.*; published by IEEE, 2021 [28].

As an emerging technology research field over the past decade, optical fluid is very suitable for the biochemical analysis of small volume analytes. Combined with traditional detection methods, optical microfluidics can accurately control, operate, and monitor the analysis process in real time. For example, Sampad et al. developed an optical flow control platform integrated with FPGA, which was used to detect and analyze single-labeled biological particles in the flow while extracting the target concentration and other experimental parameters (Figure 4) [28]. The real-time implementation by the customized Verilog program processes the photon counting of the signal in three parallel blocks, thus eliminating the time limit of the serial processing scheme. This method is proved by real-time analysis of fluorescent nanorods and single bacterial plasmid DNA. Compared with the post-processing analysis in MATLAB, the target detection is reliable, and the accuracy is 99%. Moreover, the real-time detection of each target can accurately determine the clinically relevant concentration between 3.4 × 10^4^ and 3.4 × 10^6^ per milliliter in seconds to minutes. Furthermore, the optical flow control platform can use many optical properties, including refractive index and fluorescence and Raman scattering, for signal detection alone or in combination, which is worth considering to improve the sensitivity of the detection signal.

#### 2.2.3. Digital Microfluidics Platform

**Figure 5:**
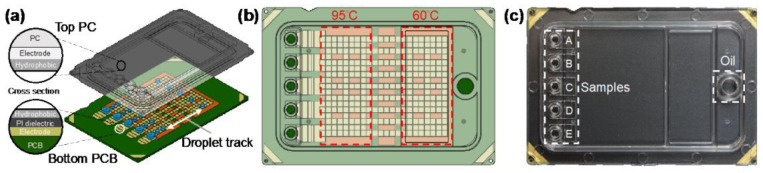
Digital microfluidic cartridge for qPCR of SARS-CoV-2 testing: (**a**) schematic diagram of the ink cartridge with the droplet trajectory; (**b**) design of the driving and reservoir electrodes; (**c**) schematic diagram of the sample inlet position. Reproduced with permission from Kuan, L, H., *Micromachines*; published by MDPI, 2022 [29].

Digital microfluidics is a disruptive technology for the design, integration, and operation of microfluidic systems based on a single droplet that utilizes precise droplet control in the microliter to nanoliter range to enable complex laboratory analyses. Digital microfluidic devices usually adopt a dielectric wetting method for droplet bioanalysis, which has the advantages of a simple structure, high sealing, avoidance of cross-contamination, and a high degree of automation. For example, Ho et al. proposed an N gene detection platform using SARS-CoV-2 based on digital microfluidics technology (Figure 5) [29]. N1 and N2 primers and probes were used, and the chip integrated electrical, thermal, and optical modules to achieve uniform temperature control and ideal fluorescence reading. The reagent volume of this platform is only 1.5 uL, which is more than 13 times smaller than the traditional desktop PCR instrument, but the amplification performance is not affected, which is comparable to that of the desktop PCR instrument.

According to the different driving forces of the fluid, we briefly introduce the principles, advantages, and improvement directions of paper-based microfluidic, centrifugal microfluidic, optical, and digital microfluidic platforms. Compared to traditional laboratory instruments, the development of these microfluidic platforms is clearly necessary for resource-constrained areas. However, there are still many difficulties for microfluidics to gain further adoption in the COVID-19 testing market. Firstly, most active microfluidic platforms still require a lot of power, which is not friendly to areas with power shortages. Secondly, although a passive microfluidic platform does not require an external pump, compared with an active microfluidic platform, the control ability and accuracy of a passive microfluidic system inevitably have some deficiencies. Finally, with the microfluidic pursuit of miniaturization and low cost at the same time, the performance of the system may decline, which is an important issue that must be balanced.

## 3. Detection Method for COVID-19 Diagnosis Based on Microfluidics

Microfluidics provides a platform for the automation, integration, and portability of POCT devices, improving the reproducibility and reducing costs and time by decreasing human intervention. At the same time, the assay method determines the efficiency, sensitivity, specificity, and accuracy of the overall system. Here, various assays combined with microfluidics are summarized.

### 3.1. Nucleic Acid Detection Method

SARS-CoV-2 is an enveloped positive single-stranded RNA virus with a genome consisting of 8–10 open reading frames encoding spike glycoprotein (S), envelope protein (E), membrane glycoprotein (M), and nucleoprotein protein (N), of which the RNA-dependent RNA polymerase and E and N genes have been widely used as targets for detection in various kits [30,31]. The WHO-recommended SARS-CoV-2 standard assay is divided into two steps: extracting the virus and performing RT-PCR. Although RT-PCR has high sensitivity and specificity to meet the requirements of the assay, it still has certain drawbacks that are not suitable for field detection Therefore, for the COVID-19 pandemic, better techniques to effectively control the outbreak are needed. In this paper, several microfluidic-based methods are presented to provide ideas for developing better POCT devices.

#### 3.1.1. Based on PCR

RT-PCR is the process of reverse transcription of viral RNA to synthesize complementary strand cDNA followed by PCR amplification. In order to effectively control COVID-19, large-scale RT-PCR tests have been conducted worldwide, but traditional RT-PCR is restricted to specialized laboratories and does not apply to POCT. On the one hand, conventional PCR requires expensive and bulky thermal cyclers, and the whole testing process is long and costly [32]. On the other hand, RT-PCR is susceptible to inhibitors within body fluids and requires specialized personnel to purify the samples before amplification. Finally, qPCR is a “semi-quantitative” technique that calculates the content of the target sequence before the sample reaction through a linear relationship between the Ct value of the sample and the logarithm of the starting copy number of the sample with a standard curve, which requires standards, and the efficiency of the PCR can affect the accuracy due to the influence of the standard curve and amplification bias. Therefore, researchers have made great efforts to simplify the operation and equipment and improve the accuracy. The following aspects are described from the combination of PCR and microfluidic platforms to simplify the procedure or improve the analytical performance.

##### Combining with Microfluidics to Simplify the Steps and Reduce the Detection Time

The combination of PCR and microfluidics improves the above drawbacks. Due to the small volume and low heat capacity of the required sample, the heating and cooling are fast enough to achieve PCR cycling by using a simple resistive microheater for spatiotemporal thermal cycling [33,34], which can avoid the use of bulky thermal cyclers, effectively shorten the assay time, and greatly reduce the volume of samples and reagents, solving the reagent shortage [35]. Yang et al. proposed a highly integrated microfluidic portable nucleic acid detection system for the detection of SARS-CoV-2 by adding a resistive heating element to achieve the control of the PCR cycle temperature, and the whole process takes only 10 min [36]. Moreover, to simplify the detection process, this study used saliva samples directly for detection, which has the advantages of easy self-collection, less patient discomfort, and ease of use compared to pharyngeal and nasal swabs. Additionally, Kang et al. proposed a chip that combines microfluidic PCR with a collection of nano-plasmon resonance sensors [37]. The chip consists of a gold nano-based glass column and a microfluidic channel containing an array reaction chamber, a vacuum cell, and a gas barrier, which contains a permeable layer and a gas permeation layer that helps to achieve a bubble-free environment for real-time quantification and prevents solution volatilization at high temperatures. Nano-plasma heating and thin samples enable the uniform vertical distribution of photothermal heat in a small volume, and the large surface area of the plasma nanopillar array facilitates rapid cooling, resulting in ultrafast photothermal cycling. The platform detects SARS-CoV-2 in 5 min with an LOD of 259 copies/uL, demonstrating the full potential of rapid virus detection.

##### Combining with Microfluidics to Improve the Automation, Accuracy, and Sensitivity

In addition to the long time and complicated procedure, the need for professional personnel is also an important factor that makes PCR technology limited to the laboratory. Huang et al. reported an automated microfluidic chip-based PCR array system that sequentially integrates sample lysis, nucleic acid extraction/purification, and real-time PCR processes on the same chip, with the entire process taking less than 90 min, and up to 21 targets can be detected and differentiated in a single application [38]. The system preloads all detection reagents onto the chip, minimizing manual operation by the user and, thus, increasing the accuracy. This automated system uses small amounts of reagents and samples, has low cost and low contamination, and enables multiplexed analysis, satisfying its use in a field environment.

**Figure 6:**
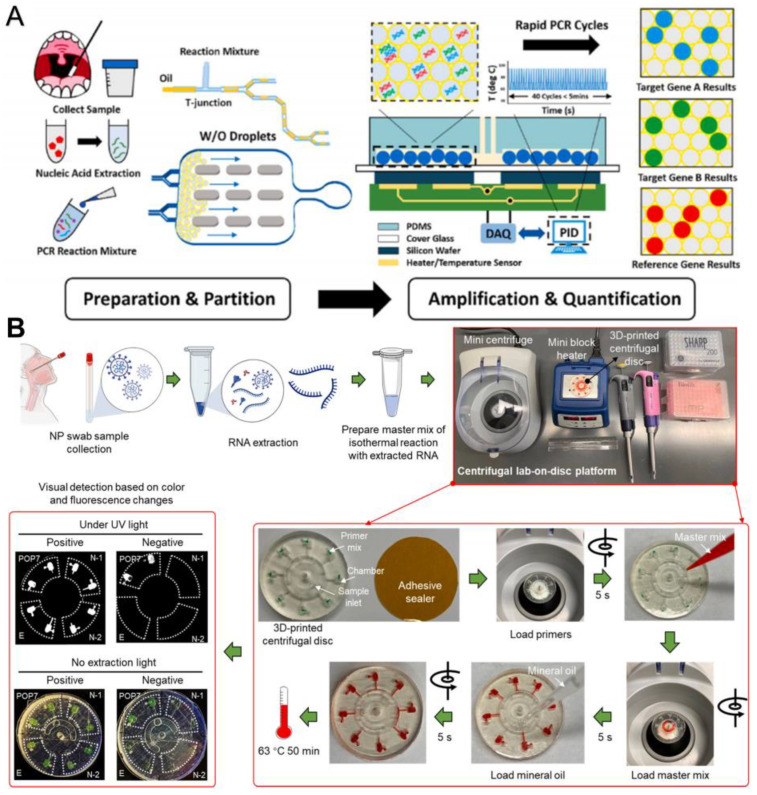
Microfluidic laboratory based on PCR assay. (**A**) Workflow of rapid digital PCR. Reproduced with permission from Yin, H., *Biosens. Bioelectron.*, published by Elsevier, 2021 [39]. (**B**) Procedures of the monolithic, 3D-printed lab-on-disk platform for the rapid, multiplexed detection of SARS-CoV-2. Reproduced with permission from Huang, E., *Anal. Bioanal. Chem.*; published by Elsevier, 2021 [40].

Yin et al. reported a combined ddPCR and rapid PCR technique, which uses a microfluidic device to generate, split, and store droplets as independent reactors for amplifying target nucleic acid sequences [39]. Two specific targets, ORF1ab and N gene, and two internal reference genes, RNase P, can be detected simultaneously, with significant positive signals detected within 115 s and accurate quantitative results provided within 15 min. This is a more accurate and rapid quantification method than microfluidic qPCR (Figure 6A). The 3D-printed disk-based microfluidic platform proposed by Ding et al. can simultaneously detect two N genes (N1 and N2 genes), the E gene, and an internal control gene, POP7; the whole process from sampling to detection takes less than 70 min, and 100 copies of SARS-CoV-2 can be detected at one time by visual observation based on the color and fluorescence changes of the disks (Figure 6B) [40].

#### 3.1.2. Based on Isothermal Amplification Technology

The main difference between isothermal amplification technology and the widely used PCR technology is whether the temperature changes. In PCR, repeatedly changing the reaction temperature affects the role of the reagents, while isothermal amplification technology amplifies DNA at a constant temperature and guides strand amplification under the action of multiple enzymes, significantly reducing the complexity of the device while also significantly reducing the amplification time due to the reduction of the temperature raising and lowering process. Therefore, compared to PCR technology, isothermal amplification technology is more suitable for POCT due to the advantages of a simple device and rapid detection. The advantages can be further expanded when combined with microfluidic technology. Common specific isothermal amplification methods include LAMP [41], rolling circle amplification (RCA) [42], cross-priming amplification (CPA) [43], strand displacement amplification (SDA) [44], nucleic acid sequence-based amplification (NASBA) [45], and RPA [46]. Among them, LAMP, RCA, and RPA have been studied in combination with microfluidic techniques for the detection of SARS-CoV-2.

LAMP, as a typical isothermal nucleic acid amplification technique, has been of interest to scholars since its emergence, and at present, it has occupied most of the market for isothermal amplification techniques. However, LAMP also has some disadvantages. The rapidity of LAMP amplification is both an advantage and a disadvantage; it leads to a high concentration of end products and a much higher probability of cross-contamination, resulting in the appearance of false positives [47,48]. Analogous to LAMP is RPA amplification. RPA is a multienzyme reaction system. It is rapid and can be completed within 5–30 min [49]. However, the reaction is usually performed by manually adding the template, primers, and buffer to tubes containing lyophilized enzyme, and for raw clinical samples, nucleic acid extraction is also required to improve the purity to reduce false negatives. Therefore, the RT-RPA process requires multiple manual operations and complex equipment. The researchers have also introduced another isothermal amplification method to address this lack of accuracy: RCA. The advantages of RCA is that the primer design is relatively simple and does not require too many primers for initiation; therefore, compared with LAMP, RCA is less likely to produce an aerosol, causing false positive results, and, additionally, the amplification product single-stranded DNA of RCA can bind directly to the probe to achieve signal amplification [50]. However, compared with other isothermal amplification techniques, RCA requires a relatively long time, phosphorylated DNA is required for ring construction, and the operation is more complicated. The excess lock-in probes and template DNA in the reaction system will produce strong background signals, which will affect the detection limits. Microfluidics shows the potential to address these limitations.

##### Combining with Microfluidic to Improve the Accuracy

**Figure 7:**
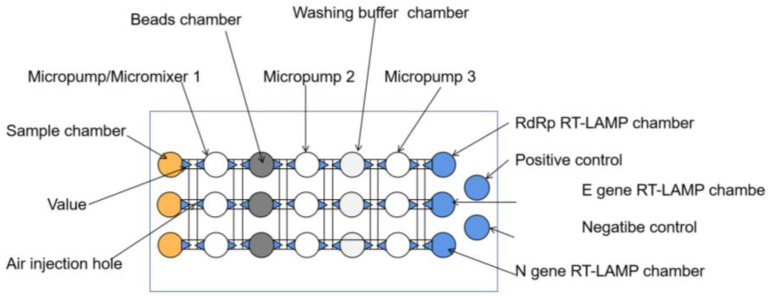
Schematic of the main components of the microfluidic chip [53].

Microfluidics can be used as a platform for multiplex biochemical reactions, immobilizing lyophilized enzymes, primers, and sensing elements onto microfluidic chips with integrated portable heaters (e.g., hand warmers) and readers, minimizing manual operations, allowing amplification procedures to be completed in a closed microarray reaction chamber, reducing cross-contamination and leakage, and reducing false positives [51,52]. For example, Jhou et al. proposed a highly integrated microfluidic platform consisting of a microfluidic chip, a temperature-controlled module, and a flow-controlled module, including the entire process of virus lysis, RNA extraction, and RT-LAMP, to automatically detect three viral genes, E, N, and RdRp, of SARS-CoV-2 in 90 min with an LOD as low as 200 copies/uL (Figure 7) [53]. This fully automatic platform minimizes manual handling, with the samples and reagents delivered and mixed by a pneumatic, membrane-based micropump and an optical detection module that collects signals every minute during RT-LAMP. The entire process is performed in a sealed microarray reaction chamber, reducing contamination generation during amplification and providing greater accuracy than traditional RT-LAMP. Similarly, Han et al. proposed a portable genetic analyzer prototype that integrated the whole process of RNA purification, RT-LAMP amplification, and real-time fluorescence detection [54]. Primers, enzymes, and reaction buffers are fixed in the reaction chamber by drying or freeze-drying techniques, and washes and eluates are automatically shunted into multiple shunt chambers after a single injection to enable the simultaneous detection of multiple samples. The platform integrates multiple units on a single centrifuge tray and can detect ten samples simultaneously in 90 min for less than USD 3 per test. In addition, the team of Soares developed an integrated modular microfluidic platform for centrifugal disks that is suitable for any smartphone readout results, with a total processing time of less than 1 h [55]. The centrifugal platform costs less than USD 250, and a single assay costs USD 2, which further reduces the cost and assay time, moreover, providing a reference solution for developing countries and poor regions. In this microfluidic platform, the researchers also introduced a multifunctional agarose bead strategy to improve the signal transduction after LAMP by eliminating the internal background of primer–dimer interactions during the insertion of fluorescent dye, which can significantly improve the resolution between positive and negative. In the same way, Liu et al. demonstrated the potential to address these methods by integrating RT-RPA with lateral flow (LF) test strips on a microfluidic chip to establish a visual method for the rapid and sensitive detection of COVID-19 [56]. The RT-RPA reaction reagent, sample, and running buffer are mixed and then sent to the LF test strip, where the naked eye observes the results. All of these steps are encapsulated in a microfluidic chip, which reduces the need for manual steps, such as incubation, buffer mixing, and LF test strip detection, and greatly reduces the potential for exposure to aerosol contaminants.

##### Combining with Microfluidics to Improve the Sensitivity

**Figure 8:**
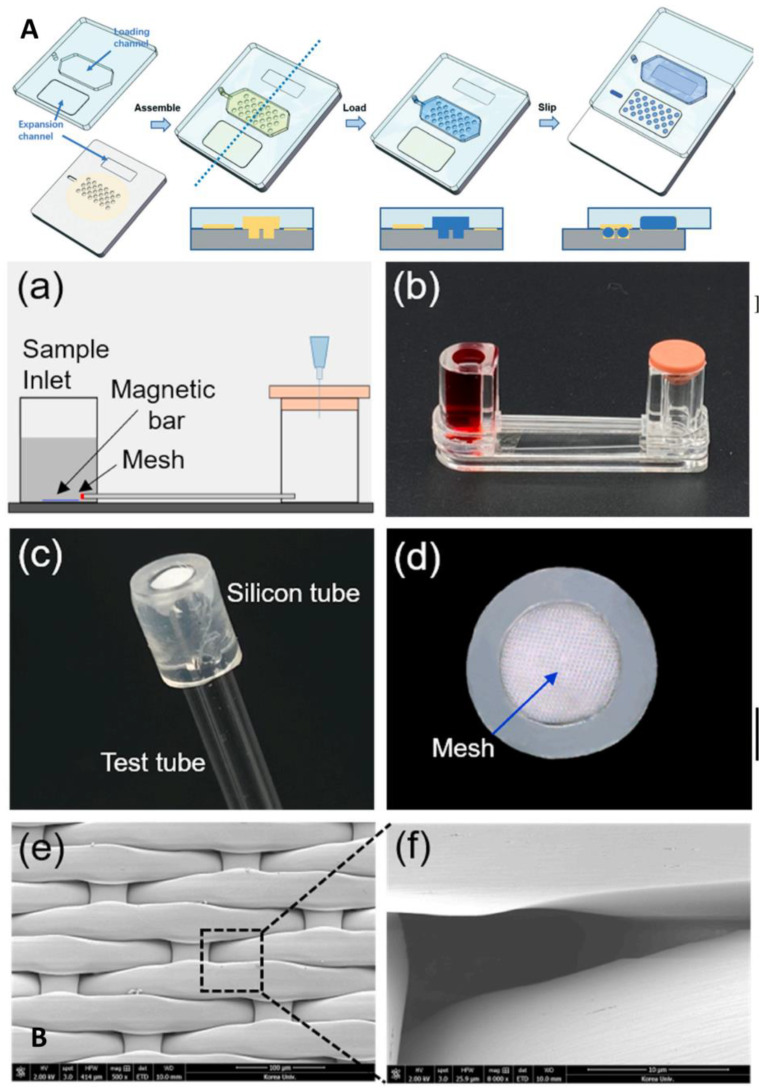
Microfluidic laboratory based on isothermal amplification technology. (**A**) Schematic demonstrating the da-Slip Chip for the slip formation of droplets at a high density. Reproduced with permission from Lyu, W., *Lab Chip*, published by Royal Society of Chemistry, 2021 [57]. (**B**) Schematics depicting the use of DNA hydrogel formation on microfluidic pores to detect SARS-CoV-2: (**a**) experimental setup; (**b**) experimental apparatus; (**c**) flexible silicon tube bonded to the nylon mesh and glass tube; (**d**) front view of the silicon tube that prevent leaks; (**e**) scanning electron image of the nylon mesh; (**f**) scanning electron image of a single micro-hole of the nylon mesh. Reproduced with permission Kim, H. S., *Biosens. Bioelectron.*, published by Elsevier, 2021 [58].

From these research results, it can be seen that isothermal amplification combined with a microfluidic platform integrates a fully automated analysis system with a high degree of containment, minimizes the intensive labor of professionals, effectively reduces experimental contamination, and further reduces the time and cost but still suffers from the same lack of sensitivity as conventional isothermal amplification. Lyu et al. proposed a droplet array sliding chip that can generate 21,696 0.25 nL droplets for use as reaction zones, with a total reaction solution of 5.4 uL and an LOD as low as 185 copies/mL, which is far below the sensitivity requirement of 500 copies/mL for corporate products (Figure 8A) [57]. The droplet array sliding chip also solves the problem of precise the micro-alignment required by conventional sliding chips by having a high-density array of wells on one plate and a liquid loading channel on the other. After the reagent is loaded into the device and enters the microwells, the two plates slide the loading channel away from the microwells, and the microwells are covered by the broad expansion channel, forming a droplet array. This approach simplifies device design while significantly increasing the number of partitions, greatly increasing the overall throughput and flexibility of digital LAMP. Kim et al. described an innovative microfluidic system that amplifies DNA hydrogels by RCA to achieve rapid and ultrasensitive SARS-CoV-2 detection in 5–15 min, with an LOD as low as 3 aM, solving the problem that RCA technology cannot achieve POCT detection (Figure 8B) [58]. The microfluidic system consists of a sample chamber, glass tube, nylon mesh for the conjugate padlock probe, and waste chamber with a rubber cap. The rubber cap is sealed so that the sample solution cannot flow into the waste chamber, and when pathogens are present in the sample, they connect to the probe on the nylon net, forming a DNA hydrogel to block the mesh path in the process. Thus, when the cap is exposed to the atmosphere, the positive sample cannot move to the waste pool. In contrast, the negative sample fluid can move smoothly and can be quantified by monitoring the fluid movement and the time it takes to reach the other end of the tube. Although this study was not conducted using clinical samples, for the time being it still provides a direction to the POCT detection of SARS-CoV-2.

In conclusion, in the context of detecting SARS-CoV-2, we analyzed the research on the combination of isothermal amplification technology and microfluidic platforms and found that experts focused their attention in two directions: automated detection and digital detection. The former gives full consideration to the major advantages of microfluidic platforms, including the functional integration, miniaturization, and portability of the device, running the whole process in a closed tiny chip, solving the problem of easy contamination and false positives inherent in LAMP and RPA technology. The latter further improves the sensitivity of the assay. However, no good solution has been proposed for the other major reason why LAMP technology cannot replace PCR technology: the restricted throughput due to the difficulty of the primer’s design. The combination of microfluidics and isothermal amplification has shown excellent performance in terms of rapid, low cost, automation, and high throughput; however, microfluidic-based isothermal amplification still has some challenges to address [59]: (1) highly concentrated buffers have the potential to cause greater resistance in the channel; (2) the elicitor needs to be prestored on the chip for nucleic acid testing integration. Therefore, these issues need to be addressed in the future by optimizing the design of the primer and fluid handling. In addition, it is worth noting that the optimal reaction temperature of RPA is 37 °C. Integrating RPA-based microfluidic platforms with electronic wearable devices for long-term nucleic acid detection is also a promising research direction [60,61].

#### 3.1.3. Based on CRISPR

CRISPR/Cas is a new powerful gene editing system, which can accurately identify and cut specific DNA and RNA sequences. Upon recognition of the target sequences, certain CRISPR/Cas systems, including homologs of Cas9, Cas13, Cas12a, and Cas14, exhibit indirect nonspecific catalytic activity that can generate fluorescent signals by degrading labeled nucleic acids, showing great potential for rapid and field-deployable nucleic acid detection [62,63], whereas its accuracy and sensitivity need further systematic validation in clinical work, with more attention to false-positive and false-negative problems.

##### Combining with Microfluidics to Improve the Degree of Automation and Reduce Cost

**Figure 9:**
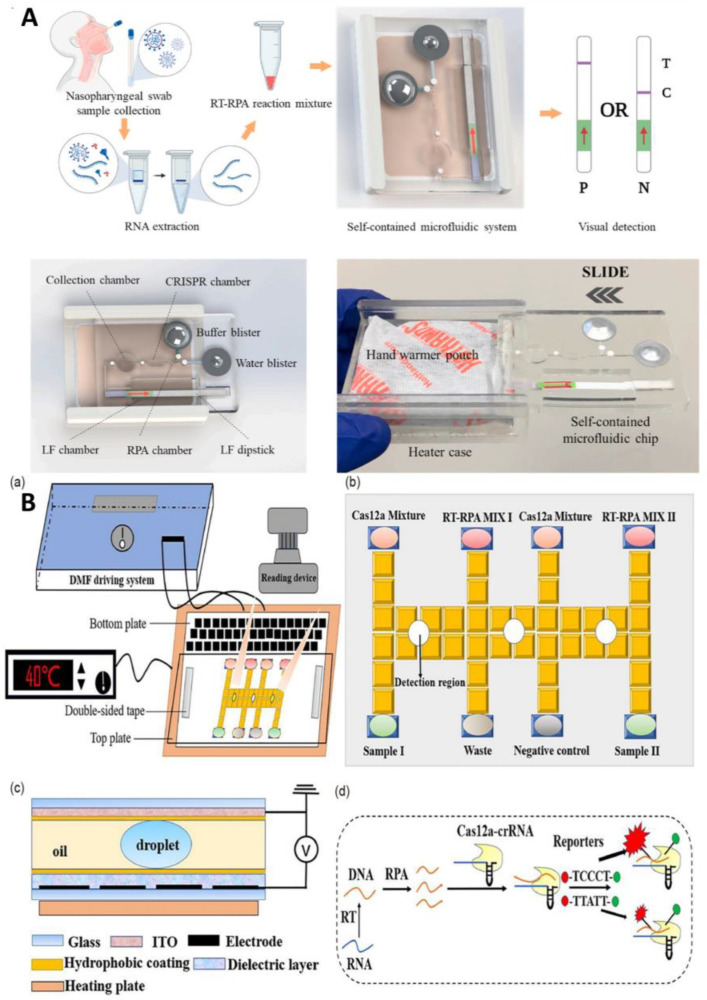
(**A**) Schematic diagram of a self-contained microfluidic system for the detection of SARS-CoV-2 in clinical samples. Reproduced with permission from Li, Z., *Biosens. Bioelectron.*, published by Elsevier, 2022 [64]. (**B**) Schematic drawings of an automated nucleic acid detection platform using digital microfluidics with an optimized Cas12a system. (**a**) Illustration of the RCD platform. (**b**) Plan view of. (**c**) Side view of the chip. (**d**) Schematic illustration of the RPA-Cas12a-crRNA recognizing target and cleaving the reporter. Reproduced with permission from Sun, Z., *Sci. China Chem.*, published by Springer, 2022 [66].

A number of researchers are working on combining microfluidics with CRISPR to improve their capabilities in the field, adding automation to the advantages of rapid detection. For example, Li et al. proposed a CRISPR-based platform for the detection of SARS-CoV-2 that integrates RPA isothermal amplification, CRISPR lysis, and lateral flow analysis on a microfluidic chip using a low-cost hand-warming bag as a heating source to provide a suitable temperature environment for RPA and CRISPR [64]. When the extracted RNA virus is added to this sealed chip, it first enters the isothermal amplification chamber for RT-RPA amplification. Then, the amplified DNA template enters the CRISPR reaction chamber, where it specifically binds to the CRISPR-Cas 12 enzyme-gRNA complex and is cleaved by the CRISPR-Cas12a enzyme, releasing carboxy fluorescein and biotin molecules. When the cleaved product is mixed with diluent, it comes to the sample pad of the LF test paper for LF analysis. The platform has good analytical performance and can detect 100 copies of SARS-CoV-2, with a 94.1% sensitivity, 100% specificity, and 95.8% accuracy, achieving instrument-free detection without cold chain and with easy transportation and operation, and no contamination, showing great potential in the rapid detection of COVID-19 in POCT (Figure 9A). In addition, Ramachandran et al. proposed a novel microfluidic detection method that combines isotachophoresis and CRISPR-Cas 12 [65]. Electric field-mediated microfluidic isotachophoresis involves nucleic acid extraction followed by off-chip RT-LAMP with amplification and then on-chip amplification products to activate CRISPR-Cas 12 for cleavage to achieve fluorescence detection of the N, E, and RNase P genes. The platform requires only 0.2 uL of reagent to complete the CRISPR reaction, which is more than 100 times lower than traditional analysis and enables the automated extraction of nucleic acids without requiring centrifugation and other steps. However, unfortunately, it also requires off-chip RT-LAMP and can only perform qualitative analysis, but it still provides us with a low-cost and rapid detection idea.

##### Combining with Microfluidics to Improve the Sensitivity and Throughput

Diagnostic tools developed based on the CRISPR system have the advantage of speed, but unfortunately they are not sensitive enough to match the “gold standard” of clinical testing—RT-PCR. Therefore, improving the sensitivity of CRISPR diagnostic technology is the key to its clinical application. Another scholar combined an optimized platform based on RPA-CRISPR with digital microfluidics to establish an automated rapid detection method for COVID-19 (Figure 9B) [66]. Digital microfluidics drives the movement, merging, and separation of individual droplets and the flow of droplets in a sealed chip for efficient mixing, thus enabling reactions to consume less time and reagents, allowing the assay to be completed within 30 min. The optimized platform for RPA-CRISPR optimizes the probe to produce the maximum fluorescence and increase the amplification signal.

The microfluidic combinatorial array reaction platform for multiplexed nucleic acid assessment proposed by Ackerman et al. paired assay reagent droplets with amplification sample droplets in a microtrap array and tested each sample with each CRISPR RNA (crRNA) in duplicate [67]. The combination of microfluidic combinatorial array reaction and Cas13 assay enables the detection of over 4500 crRNA target pairs on a single array. This high throughput and multiplex ability facilitate the transition from the single to full detection of large samples.

#### 3.1.4. Other Nucleic Acid Detection Methods

PCR and isothermal amplification are currently the most common detection methods, especially PCR technology, which occupies the majority of the domestic market due to the fact of its small technical barriers. Upon investigation, it was found that there are also some other methods implemented to detect SARS-CoV-2 in combination with microfluidics. These methods also provide a new idea for simple and sensitive virus detection. Zhang et al. proposed a direct RNA detection method based on the combination of a DNA-RNA-DNA probe and a microfluidic platform, which was performed by genome replication using DNA polymerase on DNA template with dNTP extended RNA primers, generating signals by the enzyme and detecting chemiluminescent signals from the chip with a charge-coupled device camera [68]. This method is sensitive, has few steps, can detect degraded RNA within 20 min, and the chip can be reused at least three times, because the immobilized probe is bound to washable target RNA. Similar to the use of molecular hybridization technique is an extraction-free and amplification-free high-sensitivity SARS-CoV-2 detection method based on a microfluidic platform with graphene and poly-L–Lysine materials, proposed by Chu [69]. Each detection unit of the platform is independent of the other, preventing cross-contamination between samples, so the target signal can be accurately detected. The DNA probe hybridizes well with the target RNA, with good specificity, and greatly reduces the pseudo-amplification due to the omission of a target sequence copy; in addition, the whole process does not involve any enzymes and expensive reagents. This method can detect SARS-CoV-2 in 40 min with an LOD of 600 copies/mL and at a cost of less than USD 1 per test.

Moreover, Zhao et al. developed an electrochemical system integrating reconfigurable enzyme-DNA nanostructures, which integrates multiple responsive molecular nanostructures to convert target-induced molecular activation into an enhanced electrochemical signal with an LOD of 7 copies/uL [70]. It can completely detect complex biological backgrounds in approximately 20 min at room temperature. Table 1 summarizes the microfluidic assays used to detect SARS-CoV-2 nucleic acids.

### 3.2. Immunoassay

In the early stages of the epidemic, nucleic acid testing was used as an important criterion to diagnose COVID-19. The effective testing of symptomatic patients helped us understand the characteristics of patients with COVID-19, grasp the speed and extent of transmission, and develop targeted plans for managing the epidemic. However, as the epidemic developed, it was found that asymptomatic patients and patients in the incubation period pose a great inconvenience to the management of the epidemic; therefore, we need to stop the spread of the epidemic by expanding the scope of nucleic acid testing, tracing close contacts, and isolation control. Unfortunately, nucleic acid testing requires professional personnel in the laboratory and requires a certain turnaround time to obtain the results; consequently, conducting the rapid screening of suspected patients and close contacts has become a key concern. Therefore, the rapid antigen test, which is easy to operate and requires only simple training, is expected to achieve home testing, and the results can be obtained in 15–20 min, and it is also gradually gaining attention. After the resumption of work and production, testing methods that are complemented by antigen testing on top of nucleic acid testing are even becoming a new testing strategy to improve the detection rate of positive patients and promote the recovery of social activities. With the rise in vaccination rates and the introduction of specific treatments, the detection and quantification of antibodies are being used to determine whether patients have an immune response. Viral invasion results in the production of large amounts of immunoglobulins and antibody testing in this state can determine IgG and IgM levels for monitoring purposes. In the above, we summarized some nucleic acid assays for COVID-19 in combination with microfluidics. Next, some protein (antigen and antibody) assays in combination with microfluidics will be described.

#### 3.2.1. Labeled Immunoassay

##### Fluorescence Immunoassay

Immunofluorescence assay is a method that combines antigen–antibody-specific binding with immunolabeling techniques. It is one of the earliest labeled immunofluorescence techniques that used fluorescein as a marker to label antigens or antibodies without affecting their immunological properties for the detection of antigens or antibodies and usually includes direct and indirect immunoassays. The main fluorescent immunoassays on the market are presented as lateral flow chromatography strips (LFA). The inherent limitations of LFA, such as high false positive rates (including the visual interpretation and low sensitivity) [71], limit its applicability in pressing global problems such as SARS-CoV-2, and the combination of fluorescent immunoassays with microfluidic techniques offers some solutions.

##### Combining with Microfluidics to Improve the Sensitivity and Throughput

**Figure 10:**
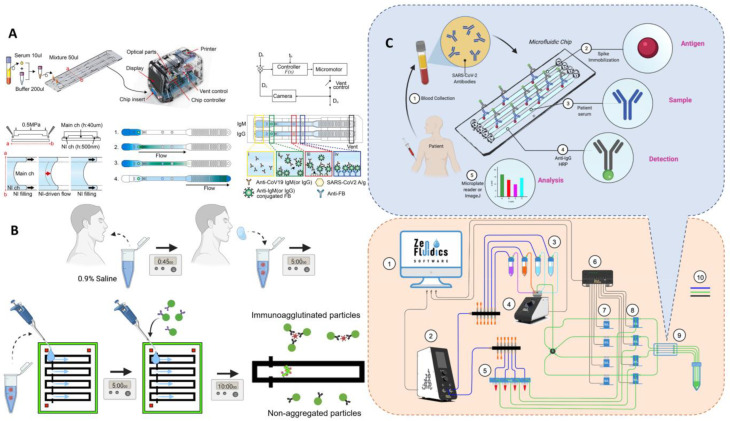
Microfluidic laboratory based on labeled immunoassay. (**A**) The assay progress of SARS-CoV-2 detection from clinical saline gargle samples. Reproduced with permission from Breshears, L. E., *PNAS Nexus*, published by Oxford Academic, 2022 [72]. (**B**) Schematic diagram of the microfluidic quantitative detection of SARS-CoV-2. Reproduced with permission from Lee, J. H., *Biosens. Bioelectron.*, published by Elsevier, 2021 [75]. (**C**) Schematic of automated ELISA on-chip for the detection of anti-SARS-CoV-2 antibodies. Lower right corner: (1) software to control all devices; (2) flow control unit; (3) ELISA reagents; (4) 12/1 bidirectional microfluidic rotary valve; (5) serum samples; (6) microfluidic valve controller; (7) pinch valves; (8) 3/2-way switching valves; (9) microfluidic device; and (10) connections between components and reservoirs (color code: blue is for pneumatic connections, green is for fluid connections, and black is for electrical connections). Reproduced with permission from Gonzalez-Gonzalez, E., *Sensors *, published by MDPI, 2021 [78].

In view of the insufficient sensitivity of fluorescence immunochromatography in the market and its inability to perform high-throughput detection, many researchers have conducted a lot of work. Lee et al. proposed a quantitative microfluidic assay with both rapid in situ characteristics similar to LFA and quantitative characteristics similar to ELISA for monitoring the immune response of patients in different clinical settings [72]. The key to this method is the introduction of two technologies: nanointerstice filling and digital fluid control. NI fills the sample liquids quickly and reliably without any hydrophilic surface treatment, allowing the main channel to be filled with stable samples even after long-term storage and, subsequently, using digital flow control to regulate NI-driven fluids (Figure 10A). Rodriguez-Moncayo reports a semi-automated high-throughput microfluidic device that can assess antibody responses to four SARS-CoV-2 antigens in up to 50 serum samples with a sensitivity of 95% and a specificity of 91% [73]. The four antigens are SARS-CoV-2 stinging protein (S), S1 subunit (S1), receptor-binding domain (RBD), and nucleocapsid protein (N). In addition, Lin et al. proposed a microfluidic platform that can simultaneously detect SARS-CoV-2 antigens and antibodies [74]. The platform integrates a homemade portable fluorescence detector and a microfluidic detection chip, which combines centrifugation, fluorescence detection, and result display functions and takes only 15 min from analysis to result acquisition, providing a useful tool for the timely screening of potentially infected patients and monitoring and prevention of epidemics.

##### Combining with Microfluidics to Simplified the Step

In addition to efforts to improve the sensitivity, many researchers are working to further simplify the detection procedure and reduce the discomfort of sampling. For example, Breshears et al. used salt-containing mouthwash sampling, loaded into a waxed paper-based microfluidic chip, and then added fluorescent particle-labeled antibodies mixed by capillary action in the chip, and positive samples with target antigens were immuno-agglutinated [75]. Finally, a smartphone-based fluorescence microscope is used to image and calculate the total area of immuno-agglutinating particles on the microfluidic channel. The innovation of this study is that no complex pharyngeal or nasal swab sampling is required, and the test can be performed using only salt-containing mouthwash, reducing the discomfort of sampling and the risk of infection that can be caused by staff sampling (Figure 10B). Similarly, a sampling method was investigated by Swank [76], using a microarray robot to dispense serum onto the epoxy-coated glass slides. All samples are analyzed for anti-spike antibodies on a chip, and the samples are collected and transported using glucose test strips, which can be easily and conveniently used in one’s own home with a simple prick of the finger and then sent via regular mail. The collected blood equipment is sent to a central laboratory that analyzes the sample for biomarkers and returns the test results to the individual via mobile information network or mail without special biosecurity requirements. Except for the testing of patients, Kim et al. proposed a system that can directly detect SARS-CoV-2 in air droplets/aerosols without the need for additional air samplers and longer sampling times [77]. The samples are passively collected on the μPADs without the use of any collectors, pumps, fans, or filters. A suspension of antibody-coupled submicron fluorescent particles is then added to the paper microfluidic chip to induce the onset of the immune response thereby causing the fluorescent particles to aggregate. Furthermore, the study produced a smartphone-based fluorescence microscope at a very low cost that can quantify the extent of fluorescent particle aggregation directly in the phone to confirm the presence of viruses in the air.

##### ELISA

ELISA is a highly sensitive immunological experimental technique combining antigen–antibody specific reaction and the efficient catalytic effect of enzymes on substrates. During the determination, the sample comes into contact with the antigens–antibodies on the surface of the solid-phase support, the free sample is separated by washing, and qualitative or semi-quantitative analysis can be performed according to the color presentation, which has the advantage of high sensitivity. However, ELISA has many steps, is a complex operation, is time consuming, and is easily contaminates; thus, there is still a long way to go if it is to be applied to field analysis.

To solve ELSIA’s complex operational problems, Gonzalez et al. developed an automated ELISA on a chip in which the S protein is immobilized on the chip. A serum sample is pumped through a microfluidic device [78]. If antibodies of S are present, they bind to the S protein and then are pumped anti-IgG-HRP to detect IgG antibodies. The analysis is performed by colorimetry, with smartphone photography and ImageJ for color intensity analysis, taking 2.5 h for the whole process. This automated microarray solves some of the problems associated with the technical limitations of existing tests, such as the need for expensive testing equipment and the labor-intensive nature of the process due to the fact of incubation and repeated washing. The automated operation minimizes manual handling, increases the reproducibility of the assay, and the microfluidic device allows for a reduction in the volume of reagents and samples, reducing costs (Figure 10C). Gong et al. used a pull-string gyroscope to separate serum from whole blood for ELISA on paper microfluidics [79]. The long arm of the foldable paper-based control reservoir is connected to the ring reaction zone to detect anti-RBD antibodies, and the immunoreactive signal is detected by HRP-catalyzed TMB-H_2_O_2_ solution, which can be directly photographed with a smartphone and analyzed by ImageJ. This method provides an economical and simple solution for separating serum to collect the assay signal without the need for large instrumentation, and the manufacturing cost of a single device is less than USD 5.

The long ELISA detection time is another important reason why it is not suitable for field detection; therefore, Liu et al. developed an ELISA on a chip that combines reciprocal flow immuno-conjugation on a chip with ELISA technology, solving the problem of the time-consuming ELISA by allowing for the detection of N proteins in just 5 min, with an LOD of 4.14 pg/mL, demonstrating the ability for rapid trace analysis [80].

#### 3.2.2. Unlabeled Immunoassay

Fluorescent immunoassays and ELISA are performed by labeling antigens/antibodies with fluorescent moieties or enzymes. Apart from this, microfluidic-based antigen–antibody detection methods were developed without labeling and based on changes in refractive index or current or capacitance.

**Figure 11:**
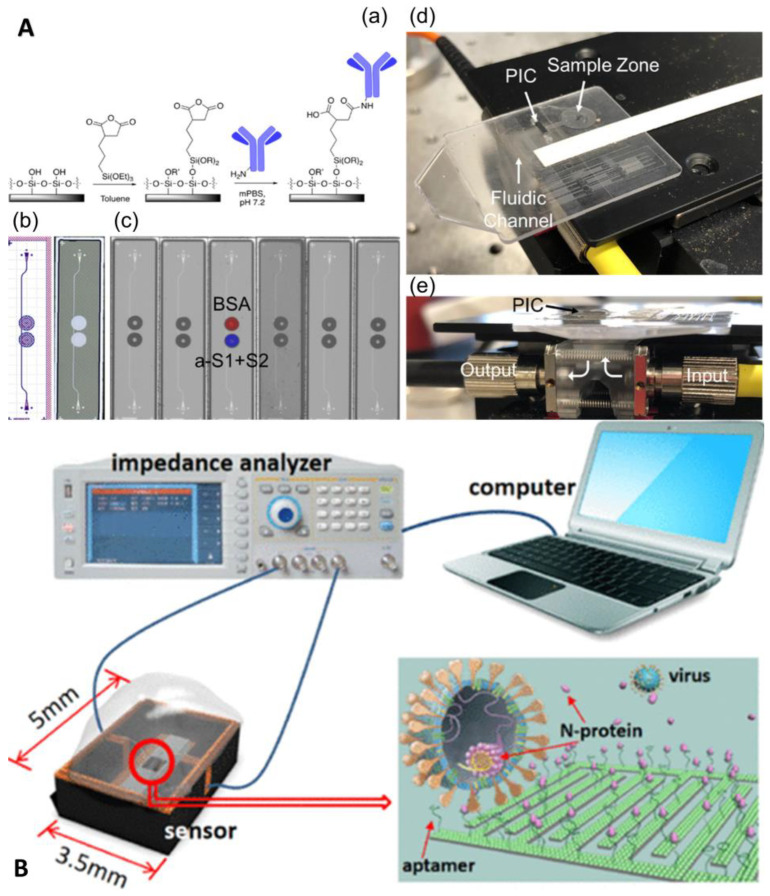
Microfluidic laboratory based on unlabeled Immunoassay. (**A**) Disposable photonic assay platform: (**a**) functionalization schematic, (**b**) GDS (graphic design system) and laser confocal image, (**c**) image of antibody/antigen solutions spotted on chips, (**d**) image of the full microfluidic/photonic chip assembly, and (**e**) sideview of the custom optical hub. Reproduced with permission from Cognetti, J. S., *Sensors*, published by MDPI, 2021 [82]. (**B**) Conceptual illustration of the sensor, measurement system, and N-protein capturing. Reproduced with permission from Qi, H., *Anal. Chem.*, published by American Chemistry Society, 2022 [84].

Funari et al. developed an optical microfluidic platform based on the principle of localized plasmon resonance, where gold nanorods are covered on a glass substrate by a two-step fabrication process of maskless lithography and gold electrodeposition [81]. When antigen–antibody binding occurs, the local refractive index changes, and this change can cause a local surface plasmon resonance (LSPR) wavelength peak shift of the gold nanorods, and the antibody concentration can be quantified by measuring the wavelength shift of the LSPR peak position on the substrate. The process can be completed in 30 min with an LOD of 0.08 ng/mL, and the chip fabrication is fast, simple, and inexpensive for large-scale generation. Cognetti et al. proposed another optical microfluidic platform based on refractive index, which combined a photonic ring resonator sensor chip with a plastic micropillar card to detect RBD-specific antibodies, and quantitative results are available in 3 min (Figure 11A) [82]. Moreover, the chip supports multiplex parallel detection, which can provide data on viral protein concentration, antibody concentration, and even cytokine concentration while keeping the cost low through chip scale production, which will be a powerful tool for infectious disease diagnosis. In addition to refracted light, the reflected light intensity can also be used for the quantitative detection of antigen–antibodies, such as a novel all-fiber optic Fresnel reflection microfluidic biosensor, developed by the team of Xu et al., based on Fresnel reflection and immunoassay principles [83]. The sensor combines an all-fiber optic system, a microfluidic chip, and a multimode fiber optic biosensor. Simple, fast, and reliable in situ detection of SARS-CoV-2 spike-in protein IgM and IgG antibodies is achieved by a simple dilution method with a one-step reaction in 7 min, with minimum concentrations of 0.82 and 0.45 ng/mL for IgM and IgG antibodies, respectively.

Label-free electrochemical immunoassay is also a very important direction. Qi et al. developed a capacitive sensor for N protein detection using solid–liquid interface capacitance as an ultra-sensitive indicator based on a specific aptamer-modified micromotor array chip with an LOD of fg/mL and a response time of only 15 s (Figure 11B) [84]. Gao et al. developed a capacitive sensor based on a graphene oxide-graphene van der Waals heterostructure-based field-effect transistor biosensor [85]. It could quantify SARS-CoV-2 stinger protein in a large dynamic range within 20 min, with an LOD as low as 8 fg/mL. Table 2 summarizes the various microfluidic-based antigen–antibody detection methods.

Currently, the epidemic prevention and control situation in China continues to improve. However, it must also be recognized that the global epidemic of COVID-19 is still at a high level, the virus is still mutating, and there is still a great deal of uncertainty about the final course of the epidemic. The prevention and control of the epidemic is still a protracted battle. Vaccines play a significant role in the long-term control of the epidemic, protecting individuals and populations. At the same time, however, given the difficulty of completely avoiding breakthrough infections, testing remains an essential tool for case identification and outbreak surveillance. The changing epidemiology has stimulated the development of multiple testing tools, and the choice of specific testing strategies in different settings is a question worth considering. Depending on the purpose of the inspection, the resources available, balancing accuracy, affordability, and the need for speed of inspection, we can quickly select the most appropriate inspection method. Finally, we summarized the performance improvement of various microfluidic features (Table 3).

## 4. Commercially Available Microfluidic Platform

At present, there are many COVID-19-specific microfluidic products on the market. These devices can detect the presence of SARAS-CoV-2 or antigens and antibodies [86,87,88,89,90,91,92,93,94,95,96,97,98,99,100]. Table 4 summarizes some current microfluidic products.

Compared with the traditional rapid PCR instrument, the microfluid-based PCR instrument has the advantages of smaller volume, faster speed, and less cross-contamination, and it can integrate the detector for one-step detection. As can be seen from the table, the nucleic acid detection time is within one hour. As for the immunochromatography technology based on microfluidic, compared with the side-flow immunochromatographic test strip commonly used in the market, first, the microfluidic technology completely abandons the membrane (the pore size of the chromatographic membrane is different and irregularly distributed. This leads to the large CV value of the chromatographic membrane products). Using the biochip as the reaction channel, the microfluidic can be accurately controlled. Secondly, microfluidic can control the accuracy, and usually the whole function of the central laboratory is integrated into a single chip, its accuracy is higher. Generally speaking, the diagnosis technology based on microfluidic has more advantages in detection performance. However, although microfluidic technology has many advantages mentioned above, it is also faced with the dilemma of high development and operating costs. If you cannot achieve “cost control”, deviate from the original intention of technology to benefit the people, it is easy to encounter high and low in the market.

## 5. Limitations and Prospects

In the context of the normalization of epidemics, the rapid, low-cost, and accurate detection of infectious diseases at the point of care is of great concern and, thus, the research and development of POCT devices has reached an unprecedented climax. The flexible combination and scale integration of multiple unit technologies on a tiny controllable platform featured by microfluidic chips have made them the first choice for modern POCT technology. This article systematically summarizes microfluidic-based diagnostic methods for COVID-19 and three common commercially available microfluidic platforms.

The Ideal POCT device needs to meet the characteristics of minimal user intervention to reduce user error, provide immediate clinical evaluation, low manufacturing, and low consumption costs to make it affordable for the user. Therefore, an ideal microfluidic device should be able to automate sample preprocessing, reaction, signal amplification, detection, data analysis, and reading. Unfortunately, we found that, for several reasons, most of the microfluidic chips are still in the proof-of-concept or prototype stage and have not played a significant role in the battle against COVID-19. First, most microfluidic devices require advanced sample pretreatment. Sample preparation is performed in Eppendorf tubes through complex centrifugation, phase separation, and kit extraction to reduce the impact of complex components in body fluids on the assay and, to a lesser extent, for the direct detection of clinical samples, often at the expense of sensitivity. Therefore, future directions for microfluidic devices for POCT may focus on how to integrate the purification step on a chip or how to use clinical samples directly for analysis while satisfying sensitivity. The second challenge in developing microfluidic assays is the speed and sensitivity of the assay, which depends on the choice of assay method and, at the same time, is an issue for outbreak prevention and control. Both nucleic acid assays and antigen- and antibody-based protein methods are emerging, and this can be solved by considering the purpose of the assay and the resources available and balancing accuracy, affordability, and the need for speed of detection. A third challenge is the issue of cost. Currently, in the industrialization of microfluidics, the technology is not very mature, and the products lack standardization; therefore, a complete industry chain has not yet been formed, and the generalization of the components cannot be achieved. In addition, the analysis chips used now are all disposable, which fails to take advantage of the reusability of microfluidic chips and further increases the cost. When the cost is affordable, microfluidic chips truly realize their social value and contribute to human health. The emerging 3D printing technology may be one way to solve the high cost. A large number of studies have shown that the use of 3D printing technology can significantly simplify the processing process of microfluidic chips, and it is also very flexible in the selection of printing materials. 3D printing is also an “experimental replication” technique that allows the entire experiment to be easily copied and perhaps shared in this way using a given design.

Although the industrialization of microfluidics still has a long way to go, we can say that the microfluidic chip is a science and technology destined to be deeply industrialized. It is worth noting that with the rapid development of mobile networks and artificial intelligence, further integration of microfluidic chips with “phones” and “Internet+” may be a major direction for future development. Although the industrialization of microfluidic still has a long way to go, we can say that microfluidic chip is a science and technology destined to be deeply industrialized. It is worth noting that with the rapid development of mobile network and artificial intelligence, the further combination of microfluidic chip with “biological mobile phone” and “Internet+” may be the general direction of future development. Microfluidic system can achieve a large amount of high-quality data, including experimental images, fluid parameters, chemical and biological detection signals and so on. However, the information contained in these data is usually not directly available. The advantage of artificial intelligence in large-scale data processing can just make up for this defect. In addition, the high versatility of signal detection and target recognition ability of artificial intelligence can reduce the dependence on specific detection equipment, reduce hardware requirements, reduce the size of equipment, and save detection costs. promote the development of microfluidic system in miniaturization and integration. With the addition of the Internet, data sharing can be realized. Finally, we sincerely hope that microfluidic technology will flourish and play a more important role in the fight against the COVID-19 epidemic, and we also hope that our report can provide a new direction for the control of the epidemic.

## Figures and Tables

**Table 1:** Microfluidic assays for the detection of SARS-CoV-2 nucleic acid.

Microfluidic Platform	Improvement	Type of Technique	Target Gene	LOD	Processing of Time	Characteristics	Reference
μPADs	High integration	CRISPR-RPA	N, S	100 copies/rnx	<60 min	Paper-based sugar valve	[23]
Centrifugal microfluidic platform	High integration	LAMP	N	NR	8 min	Automatic control of laser airborne microvalve instead of passive valve	[26]
Centrifugal microfluidic platform	Step simplification and High integration	LAMP	N, E	0.5 copies/uL	60 min	Rapid test in whole blood	[27]
Optical flow control platform	High sensitivity	NR	NR	NR	<10 min	FPGA optical flow control platform	[28]
Digital droplet microfluidic platform	High sensitivity	qPCR	N	NR	NR	Dielectric electrowetting	[29]
Sample-in–result-out integrated microfluidic platform	Step simplification and High integration	qPCR	N	9 copies/rnx	10 min	Use a saliva sample directly	[36]
Array-based microfluidic chip	Reduce detection time	PF-PCR	E	259/rnx	326 s	Pressure driven, vacuum assisted plasma nanocolumn array	[37]
Sample-in–result-out integrated microfluidic platform Multiplexing platform	High integration	qPCR	ORF1ab	1 copies/uL	<90 min	Pressure driven, parallel channel multiplexing	[38]
Digital droplet microfluidic platform	High accuracy	dPCR	N, ORF1ab	5 copies/rnx	<15 min	In situ array heater	[39]
Centrifugal microfluidic platform	High throughput and visualization	qPCR	N, E	5 copies/rnx	70 min	Centrifugal force drive, 3D printing technology	[40]
Sample-in–result-out integrated microfluidic platform	High accuracy and integration	LAMP	N, E, and ORF1ab	5 × 10^3^ copies/rnx	<90 min	Pneumatic film based micropump	[53]
Centrifugal microfluidic platform	High accuracy and integration	LAMP	N, S, and ORF1ab	2 copies/µL	<90 min	Centrifugal force drive, smartphone modularization	[54]
Centrifugal microfluidic platform	High accuracy and integration	LAMP	ORF1ab	100 copies/rnx	<60 min	Capillary action driven, multifunctional agarose bead strategy	[55]
Sample-in–result-out integrated microfluidic platform	High accuracy and integration	RPA	N	30 copies/rnx	30 min	Capillary driven, integrated lateral flow test bar, visible	[56]
Digital droplet microfluidic platform	High sensitivity	dLAMP	NR	185 copies/mL	NR	Droplet array sliding chip	[57]
A microfluidic platform based on capillary interaction	High sensitivity	RCA	ORF1ab	0.7 aM/rxn	5–15 min	DNA hydrogel	[58]
Sample-in–result-out integrated microfluidic platform	High degree of automation and integration	LFA-CRISPR-RPA	N	10 copies/rnx	45 min	warm bag as heat source	[67]
Electric field mediated microfluidic platform	High degree of automation	CRISPR	N, E	10 copies/uL	30–40 min	Electric field mediated microfluidic iso-electrophoresis for nucleic acid extraction	[68]
Digital droplet microfluidic platform	High sensitivity	CRISPR	N, ORF1ab	5.2 copies/rnx	30 min	Digital microfluidic	[69]
Array-based microfluidic chip	High throughput	CRISPR	N	aM	NR	Microporous array	[70]
Optical flow control platform	High sensitivity	Molecular hybridization	N, ORF1ab	1.0 pM	20 min	Probe DNA-RNA-DNA	[71]
Electric chemistry microfluidic	High accuracy	Molecular hybridization	N, E, and ORF1ab	600 copies/ mL	40 min	graphene and poly-lysine materials	[72]
Electric chemistry microfluidic	High sensitivity	Molecular hybridization	S	7 copies/uL	20 min	Integrated reconfigurable enzyme-DNA nanostructures	[73]

LOD, limit of detection; NR, not reported; rxn, reaction; LFA, lateral flow assay.

**Table 2:** Microfluidic immunoassays for the detection of SARS-CoV-2 antigens/antibodies.

Microfluidic Platform	Improvement	Method	Protein	LOD	Time	Test Method	Characteristic	Reference
μPADs	Step simplification	Electrochemistry	NR	NR	30 min	Electrochemical	ZnO nanowires were synthesized directly	[22]
Digital droplet microfluidic platform	High sensitivity	Indirect immunofluorescence	Anti-N	NR	<5 min	Fluorescence	Nanogap filling and digital fluid control	[72]
Array-based microfluidic chip	High sensitivity and throughput	Indirect immunofluorescence	Anti-N, S and RBD	1.6 ng/mL	2.6 h	Fluorescence	Pressure pumping, microarray	[73]
Sample in- result out integrated microfluidic platform	High sensitivity	Indirect immunofluorescence	VP	NR	15 min	Fluorescence	Smartphone	[74]
μPADs	Improved sampling mode	Direct immunofluorescence	N	10 ag/uL	20 min	Fluorescence	Capillary action, mouthwash	[75]
Array-based microfluidic chip	Improved sampling mode	Indirect immunofluorescence	Anti-S	327 pg/unit	NR	Fluorescence	Microarray robot	[76]
μPADs	Improved sampling mode	Direct immunofluorescence	Anti-N	200 pg/mL	30 min	Fluorescence	Air sample collection	[77]
Array-based microfluidic chip	High degree of automation	ELISA	Anti-S	NR	<2.5 h	Colorimetric	Smartphone	[78]
μPADs	High degree of automation	ELISA	Anti-RBD	NR	4–5 h	Fluorescence	Foldable paper-based pair	[79]
Sample in- result out integrated microfluidic platform	Reduce detection time	ELISA	N	4.14 pg/mL	5 min	Fluorescence	Reverse phase flow immunocoupling technique	[80]
Optical flow control platform	Mass production and high sensitivity	Label-free optical immunoassay	Anti-RBD	0.08 ng/mL	<30 min	Reflected light	Peak shift of local surface plasmon resonance (LSPR) wavelength in gold nanorods	[81]
Optical flow control platform	Mass production and high sensitivity	Label-free optical immunoassay	Anti-RBD	NR	3 min	Absorbance	Photonic ring harmonic oscillator	[82]
Optical flow control platform	Reduce detection time and high sensitivity	Label-free optical immunoassay	Anti-S	0.82 ng/mL 0.45 ng/mL	7 min	Reflected light	All-fiber Fresnel reflection	[83]
Micromotor array chip	High sensitivity	Label-free electrochemical immunoassay	N	fg/mL	15 s	Electrochemical	Solid-liquid interface capacitance	[84]
Electric chemistry microfluidic	High sensitivity	Label-free electrochemical immunoassay	S	8 fg/mL	20 min	Electrochemical	Graphene oxide	[85]

LOD, limit of detection; N, nucleocapsid protein; S, spike protein; VPs, unspecified SARS-CoV-2 viral proteins; NR, not reported.

**Table 3:** Improvement in the microfluidic performance.

Product	Manufacturer Name
Simplified steps	Sample collection method; capillary driven; 3D printing; paper-based microfluidic
Reduced time	Small volume and large surface area array; heating cycle in-situ heater array; digital droplet efficient mixed; centrifugal microfluidic
High throughput	Array; centrifugal microfluidic
Multiplexing	Centrifugal microfluidic; multiple closed reaction chambers
Improved accuracy	Strategy of sealing multifunctional agarose beads; closed reaction chambers
Improved sensitivity	Digital droplet; DNA hydrogel; graphene and poly 1-lysine material; E-construct enzyme-DNA nanostructure; photonic ring oscillator
Improved integration and automation	Valve; setting air cavity; nano-array; centrifugal microfluidic; pneumatic film-based micropump; warm handbag as heat source; smartphone modularization

**Table 4:** Commercially available microfluidic platforms for COVID-19.

Product	Manufacturer Name	City and Country	Type of Platform	Target	Limit of Detection	Processing Time (Minutes)	Reference
ID NOW™ COVID-19	Abbott Diagnostics Scarborough, Inc.	Illinois, USA	NEAR	RdRp gene	125 genome equivalents per mL	15	[86]
Foaming Test	Pharma Nona	Udine, Italy	POC/Near POC	N Gene	NR	1	[87]
Vita PCR™ SARS-CoV-2 Gen2 Assay	Credo Diagnostics Biomedical Pte. Ltd.	New Taipei, Singapore	RT-PCR	N Gene	30 copies/reaction	25	[88]
The Bio-Fire^®^ Respiratory 2.1-EZ (RP2.1-EZ) Panel (EUA)	Bio-Fire Diagnostics, LLC	Utah, USA	RT-PCR	S, E Gene	500 copies/L	45	[89]
Xpert Xpress SARS-CoV-2 test	Cepheid	California, USA	RT-PCR	N, E gene	NR	45	[90]
1copy COVID-19 qPCR Kit	1drop Inc	Seongnam, Republic of Korea	RT-PCR	E and RdRp gene	200 copies/L	22	[91]
Biosynex COVID-19 Ag+ BSS Rapid Test	BIOSYNEX S.A., Switzerland	llkirch-graffenstaden, France	RT-PCR	N-protein	750 TCID50/mL	10	[92]
AQ-TOP COVID-9 Rapid Detection Kit PLUS	SEASUN BIOMATERIALS	Seoul, Republic of Korea	RT-PCR	Orf1ab	1 copy/uL in single reaction	30	[93]
Novel Coronavirus (2019-nCoV) RT-PCR Detection Kit (Lyophilized)	Shanghai Chuangkun Bitech Inc.	Shanghai, China	RT-PCR	S	500 copies/uL	70	[94]
SARS-CoV-2 IgM/IgG Antibody Assay Kit	Zybio Inc.	Chongqing, China	Colloidal Gold method	NR	NR	15	[95]
Lucira COVID-19 All-In-One Test Kit	Lucira Health, Inc.	Delaware, United States	RT-LAMP	N Gene	1100 TCID50/mL	30	[96]
Respiratory Virus Nucleic Acid Detection kit	CapitalBio Technology	Beijing, China	Isothermal amplification	N	NR	90	[97]
Omnia SARS-CoV-2	Qorvo Biotechnologies	Minnesota, USA	Antigen immunoassay	Protein	NR	~20	[98]
LumiraDx SARS-CoV-2 Ag test	LumiraDx	London, United Kingdom	Antigen immunoassay	N-protein	32 TCID50/mL	12	[99]
Sampinute COVID-19	Celltrion	Incheon, Republic of Korea	Antigen immunoassay	N, S-protein	NR	30–45	[100]

## Data Availability

Not applicable.
